# Social support and quality of life among chronically homeless patients with schizophrenia

**DOI:** 10.3389/fpsyt.2022.928960

**Published:** 2022-07-27

**Authors:** Jinliang Chen, Hongli Song, Shuchun Li, Ziwei Teng, Yuhan Su, Jindong Chen, Jing Huang

**Affiliations:** ^1^Department of Psychiatry, Shenzhen Hospital of Integrated Traditional Chinese and Western Medicine, Shenzhen, China; ^2^Department of Psychiatry, Fifth Ren Min Hospital of Xiangtan, Xiangtan, China; ^3^National Clinical Research Center for Mental Diseases and Department of Psychiatry, The Second Xiangya Hospital of Central South University, Changsha, China; ^4^China National Technology Institute on Mental Disorders, Changsha, China

**Keywords:** schizophrenia, homeless, non-homeless, quality of life, socio-support

## Abstract

This study aimed to describe the sociodemographic characteristics, social support received, and quality of life of chronically homeless patients with schizophrenia in China. A self-prepared sociodemographic questionnaire, the Social Support Rating Scale (SSRS), European Five-dimensional Health Scale (EQ-5D), and Eysenck Personality were administrated to 3,967 chronically homeless and 3,724 non-homeless patients from the Department of Xiangtan Fifth People's Hospital, Hunan, China, between April 2011 and October 2016. Results indicated that the homeless patients were more likely to live outside the city and be ethnic minorities compared with non-homeless patients. Although the married proportion was higher among homeless patients, they had a higher rate of being divorced or widowed. Notably, the homeless patients had higher employment rates before illness, despite significantly lower education (*P* < 0.001). Chronically homeless patients with schizophrenia showed a lower score in the SSRS (30.29 ± 7.34 vs. 26.16 ± 10.04, *p* < 0.001); they had significantly lower objective support, subject support, social support, and EQ-Visual Analog Scale, Eysenck Personality Questionnaire-Psychoticism, and Eysenck Personality-Neuroticism scores (*p* < 0.001). Homeless patients may be worse off, and could be assisted by providing accommodation, family intervention, medical services (such as pain medication), and other comprehensive measures.

## Introduction

Despite rapid economic and social development and the progress of human civilization, the living condition of homeless patients with mental disorders is a growing concern. Homelessness is a social problem in China and remains understudied (1). A large number of homeless patients with schizophrenia is now challenging for mental health care management. The major contributing factors to homelessness are domestic violence, psychiatric disorder, housing crisis, and substance misuse (2–5). A recent systematic review and meta-analysis estimated the pooled prevalence of psychotic disorders among homeless people and found a remarkably high prevalence of schizophrenia (10.29% [95%, CI: 6.44, 16.02]), which was higher in developing than developed countries (22.15 vs. 8.83%) (6).

Social factors are important considering the status of homeless patients with schizophrenia and require urgent attention and action (7–9). Social and psychosocial interventions have been proven to be effective treatments and helpful in managing long-term psychiatric disorders (10). While psychiatry has shifted its focus to a more biological approach (11, 12), social factors are important regarding psychiatric disorders relating to social deprivation, rehabilitation, and enabling social inclusion. The degree to which society is willing to accept people with mental health problems impacts their quality of life (QoL). A 10-year Chinses cohort study evaluated predictors of homelessness in patients with schizophrenia during the follow-up period. Several social-related predictors were found: poor living conditions, low income, and little support from family (13). Most studies on this topic were conducted in developed countries (7, 14, 15), no associations between social support and QoL among homeless patients with schizophrenia were reported in China.

Our study aimed to describe the sociodemographic characteristics, social support, and QoL of homeless patients with schizophrenia compared to non-homeless patients, and provide some evidence to help the government establish interventions and social support systems.

## Methods

The participants of this study were inpatients with schizophrenia from Xiangtan Fifth People's Hospital, Hunan Province. Located in the south-central area of China, Xiangtan Fifth People's Hospital is the largest specialty psychiatric hospital in the locality, with 1,200 hospital beds. The study protocol was approved by the Ethics Committees of the Fifth People's Hospital of Xiangtan (No. 20160217). All methods were performed in accordance with the relevant guidelines and regulations. Before being interviewed, written consent was obtained from all the participants and their guardians, and declarations of anonymity and confidentiality were made. Any modification was submitted to the ethics committee for discussion and approval.

### Enrollment criteria

All participants in our study met the criteria of the Diagnostic and Statistical Manual of Mental Disorders Fourth Edition (DSM-IV) for a diagnosis of schizophrenia, were aged between 15 and 60 years, were in remission after treatment, and were able to understand and complete the questionnaire. The inclusion for the chronically homeless patients with schizophrenia were those who met the DSM- IV criteria and lived without suitable or temporary accommodation according to the European Typology of Homelessness and Housing Exclusion (ETHOS) definition, such as rooflessness, houselessness, living in insecure housing, living in inadequate housing (16); being homeless for more than 1 year or ≥four separate times in the last 3 years and four combined occasions equal to at least 1 year^1^ The non-homeless patients with schizophrenia were those who met the DSM-IV criteria and had a fixed residence, usually sent to hospital by his family or himself.

### Relief treatment process for the homeless patients

Almost all patients were found by concerned locals who called the hotline 1-1-0 so the homeless may be sent to the hospital by the police or by the Rescue Shelter (the department providing help for homeless people). When homeless patients were brought to the hospital, the Police or Rescue Shelter staff filled in the rescue application form, which included the time, place, mental and physical state, and personal belongings of the homeless when they were found. They received professional treatment after entering the hospital, and doctors carefully recorded changes in the patients' conditions. Some of the survey data came from these records. After the patient's remission, and upon obtaining clear identification information, the Rescue Shelter staff return the homeless patient to their registered household. A significant number of the participants could not state their personal, family, or any identification details because of their disorders; however, most of this information was recalled after treatment and from interaction with their relatives.

### Self-prepared sociodemographic questionnaire

A self-prepared sociodemographic questionnaire was used, which covered nine dimensions: sex, ethnic group, age, and registered household [in China, Hukou usually refers to the place where they primarily lived, where the residents' parents registered their household when they were born (17)], educational levels, marriage, and employment.

### Social support rating scale (SSRS)

The SSRS was revised by Xiao Shuiyuan and has good reliability and validity for the assessment of the physical and mental health of Chinese people (18, 19). It includes 10 items comprising objective support (three items), subjective support (four items), and utilization of socio-support (three items). The scoring method uses 1–4 points for each item numbered 1–5 and 8–10, 1 point for each item numbered 6–7, and 0 points for no response. A total score of ≥20 shows a high degree of social support; the higher the score, the higher the social support. A total score of less than 20 indicates less social support. Those who score between 20 and 30 have a general degree of social support, and those who score between 30 and 40 have a satisfactory degree of social support.

### European quality of life five-dimensional scale

The EQ-5D contains simple content and shows high reliability and validity (20, 21). It is divided into two parts: the EQ-5D health description system and EQ-VAS, which can be used to evaluate health decline caused by certain diseases in patients. The EQ-5D health description system includes five dimensions: “mobility (walking about),” “looking after myself,” “doing usual activities (e.g., going to school, hobbies, sports, playing, doing things with family or friends),” “having pain or discomfort,” and “feeling worried, sad, or unhappy.” Each dimension contains three levels: no problems, some problems, and a lot of problems. To better analyze health utility in our study, we used the time trade-off method utility value conversion table to convert the five dimensions of the EQ-5D health description system into integral values. As there is no corresponding conversion table in China, the EQ-5D health index score (between −0.11 and 1.00), obtained by the Japanese integration conversion table recommended in the literature was used in this study. The healthy index score = 1 constant term was used as the standard coefficient corresponding to the different levels of each dimension, in which the constant term = 0.152; the higher the score, the better the QoL. The EQ-VAS is a 20-cm vertical visual scale, with a top score of 100 representing “the best imaginable health” and a bottom score of 0 representing “the worst imaginable health.” In this study, the actual corresponding score divided by 100 was used, that is, the EQ-VAS health score was between 0 and 1.

### Eysenck personality questionnaire

The EPQ measures traits of four personality dimensions: psychoticism (P), extroversion (E), neuroticism (N), and lie scale (L) (22).

All evaluations were conducted by specially trained psychiatrists. Uniform instructions and appropriate explanations were given for sentences that were incomprehensible to the participants, but without providing further hints. The test lasted for at least 30 min for each participant. The homeless in remission and the non-homeless completed self-prepared sociodemographic questionnaire, the SSRS, EQ-5D, and EPQ questionnaires based on the enrollment criteria. Since most of the participants' relatives were found, the veracity of the questionnaire answers was further checked.

### Statistical analysis

All the data in this study were processed and analyzed used SPSS version 25.0. Data were examined for the presence of missing values, influential values and outliers, skewness, and kurtosis. Scales and indices were tested for reliability. Kolmogorov–Smirnov one-sample test was used to measure the normal distribution of continuous variables. Categorical variables were tested by Chi-square test, and continuous variables were tested by Mann-Whitney U test in this study. Correlation between different scales were analyzed by Spearman correlation. Further regression models (stepwise) were then carried out in the participants' samples. EQ-VAS were the dependent variable, and the independent factors were sex, age, objective support, subjective support, social support, P, E, N and L. A two-tailed *p* value < 0.05 was considered significant in this study.

## Results

### Demographics and clinical characteristics

This study was conducted between April 2011 and October 2016. All the homeless patients during this period were primarily screened. A total of 4,983 homeless patients were included. Among them, 379 (7.8%) refused to participate, 334 (6.7%) did not meet the inclusion criteria, and 293 (5.9%) had incomplete data. Finally, data of 3,967 patients were included. We also included non-homeless patients with schizophrenia in similar numbers for comparison. 4,500 patients during this period were screened, 520 (11.6%) refused to participate, 127 (2.8%) did not meet the inclusion criteria, and 119 (2.6%) had incomplete data. Thus, data of 3,734 non-homeless patients were included.

The participants were mainly of Han ethnicity, accounting for 89–95.2% of the total population. The proportion of ethnic minorities among the homeless patients was higher than that among the non-homeless (11.0 vs. 4.8%, *P* < 0.001). The homeless patients were often found outside the city (87.0%) and most were migrants (58.1%), while non-homeless patients were often sent to the hospital from the place where they primarily lived (70.3%), and were mostly local residents (86.3%). Most patients were not married, with an unmarried rate of over 56.6%. Homeless patients had a higher rate of being divorced and widowed (17.7%) compared with non-homeless patients (13.7%). The employment rate of homeless patients before falling ill was higher than that of non-homeless patients (72.6 vs. 52.4%, *P* < 0.001). A large proportion of them had a low educational level (88.3% primary school education level or below among homeless patients compared with 53.6% among the non-homeless). Almost half of the participants were smokers (47.3–49.7%). The mean age of the homeless patients was 36.11 years, which was higher than the non-homeless, who had a mean age of 33.34 years. There was no significant difference in sex between the groups (*P* = 0.664) (Table 1).

**Table 1:** Comparison of sociodemographic differences between the homeless and non-homeless schizophrenia patients.

**Sociodemographic differences**	**Homeless (*****n*** = **3,967)**	**Non-homeless (*****n*** = **3,734)**	χ^2^	***P***
Sex			0.189	0.664
Male	2,565(64.7%)	2,432(65.1%)		
Female	1,402(35.3%)	1,302(34.9%)		
Age	36.11 ± 10.04	33.34 ± 11.72	11.147	0.001
Rural	3,450(87.0%)	2,624(70.3%)	321.706	0.001
HAN ethnicity	3,531(89.0%)	3,554(95.2%)	99.506	0.001
Smoking				
Smokers	1,876(47.3%)	1,856(49.7%)	4.493	0.034
Non-smokers	2,091(52.7%)	1,878(50.3%)		
Household registration			1630.739	0.001
Xiangtan	1,664(41.9%)	3,222(86.3%)		
Non-Xiangtan	2,303(58.1%)	5,12(13.7%)		
Education level			1147.656	0.001
Primary school or below	3,501(88.3%)	2,000(53.6%)		
Junior high school	2,94(7.4%)	893(23.9%)		
High school	172(4.3%)	841(22.5%)		
Senior high school or above	172(4.3%)	841(22.5%)		
Employment (Employed-before illness)			373.576	0.001
Stable job	768(19.4%)	576(15.4%)		
Temporary job	2,112(53.2%)	1,334(36.0%)		
Jobless	1,087(27.4%)	1,814(48.6%)		
Marital status			96.258	0.001
Single	2,245(56.6%)	2,518(67.4%)		
Married or remarried	1,018(25.7%)	704(18.9%)		
Divorced or widowed	704(17.7%)	512(13.7%)		

### Comparisons of socio-support, QoL, and personality between the homeless and non-homeless patients

Homeless patients with schizophrenia had significantly lower total scores on the Social Support Rating Scale (SSRS; 30.29 ± 7.34 vs. 26.16 ± 10.04, *p* < 0.001), with significantly lower objective, subjective, and social support (*p* < 0.001). The European Quality of Life Five-Dimensional Scale (EQ-5D) health index scores among homeless patients were significantly lower, although the EQ Visual Analog Scale (EQ-VAS) score was higher (Table 2). Homeless patients with schizophrenia had significantly higher scores on the Eysenck Personality Questionnaire for psychoticism (EPQ-P; 55.84 ± 9.97 vs. 52.99 ± 8.90, *p* < 0.001) and neuroticism (EPQ-N; 55 ± 11.14 vs. 49.18 ± 11.58, *p* = 0.001).

**Table 2:** Comparison of SSRS, EQ-5D, and EPQ differences between the homeless and non-homeless schizophrenia patients.

**Variables**	**The non-homeless**	**The homeless**	***Z***	***p***
EQ-5D Health description	0.74 ± 0.11	0.73 ± 0.12	−1.12	0.262
EQ-VAS	11.21 ± 0.18	14.59 ± 0.23	−12.719	<0.001
SSRS total scores	30.29 ± 7.34	26.16 ± 10.04	−25.64	<0.001
objective support	15.54 ± 3.98	15.08 ± 5.12	−3.49	<0.001
subjective support	6.98 ± 3.12	4.86 ± 4.31	−36.13	<0.001
use of social support	7.52 ± 2.04	6.48 ± 2.49	−18.93	<0.001
EPQ-P	52.99 ± 8.90	55.84 ± 9.97	−14.17	<0.001
EPQ-E	49.62 ± 9.65	46.75 ± 11.73	−11.27	<0.001
EPQ-N	49.18 ± 11.58	55 ± 11.14	−22.11	<0.001
EPQ-L	52.62 ± 8.45	52.11 ± 8.85	−2.59	<0.001

Spearman correlation analysis was performed to evaluate the relationships between homeless status and SSRS, EQ-5D, EQ-VAS, and EPQ scores. We found that homeless status was negatively associated with SSRS, EQ-VAS, EPQ-E, and EPQ-L scores but positively associated with EPQ-P and EQP-N scores, whereas it showed no significant relationship with EQ-5D health description (Table 3).

**Table 3:** The spearman correlation between the homeless, SSRS, EQ-5D, EQ-VAS, and EPQ.

**Variables**	**The homeless**	**SSRS total scores**	**Objective support**	**Subjective support**	**Use of social support**	**EQ-5D Health description**	**EQ-VAS**	**EPQ-P**	**EPQ-E**	**EPQ-N**
The homeless	1									
SSRS total scores	−0.244**	1								
Objective support	−0.034**	0.736**	1							
Subjective support	−0.352**	0.672**	0.442**	1						
Use of social support	−0.187**	0.526**	0.429**	0.563**	1					
EQ-5D Health description	−0.011	0.092**	0.071**	0.095**	0.028**	1				
EQ-VAS	−0.122**	0.210**	0.157**	0.196**	0.178**	0.446**	1			
EPQ-P	0.134**	−0.279**	−0.224**	−0.267**	−0.279**	0.185**	−0.006	1		
EPQ-E	−0.106**	0.342**	0.322**	0.219**	0.187**	0.023**	0.074**	−0.066**	1	
EPQ-N	0.209**	−0.224**	−0.150**	−0.225**	−0.090**	−0.198**	−0.088**	0.086**	−0.161**	1
EPQ-L	−0.027**	0.177**	0.103**	0.199**	0.209**	0.011	−0.089**	−0.048**	0.103**	−0.220**

** presents *p* < 0.01.

The correlations between the factors measured by the SSRS, EQ-5D, EQ-VAS, and EPQ were examined. SSRS total scores had a positive correlation with objective (*r* = 0.736, *p* < 0.01), subjective (*r* = 0.672, *p* < 0.01), and social support (*r* = 0.526, *p* < 0.01). SSRS total scores were also significantly related to EQ-5D health description (*r* = 0.092, *p* < 0.01) and EQ-VAS (*r* = 0.210, *p* < 0.01) but not as strongly as with EPQ-E (extroversion) (*r* = 0.342, *p* = 0.008 <0.01). This indicated a significant positive linear fit of the SSRS and EPQ but only a slightly positive linear fit of the SSRS and EQ-5D. In contrast, the SSRS had negative relationships with EPQ-P and EPQ-N (*r* = −0.279, *p* < 0.01, and *r* = −0.224, *p* < 0.01, respectively) (Table 3).

After controlling for confounding factors, the following variables were still significant for lower QoL (which was evaluated by EQ-VAS): being homeless (Beta = −0.156, *t* = −14.29, *p* < 0.001), female (Beta = −0.2, *t* = −19.406, *p* < 0.001), higher EPQ-E score (Beta = −0.047, *t* = −3.941, *p* < 0.001), higher EPQ-N score (Beta = −0.119, *t* = −10.195, *p* < 0.001), and higher EPQ-L score (Beta = −0.156, *t* = −13.756, *p* < 0.001). Older age (Beta = 0.219, *t* = 20.112, *p* < 0.001), more objective support (Beta = 0.283, *t* = 18.419, *p* < 0.001), more subjective support (Beta = 0.077, *t* = 5.171, *p* < 0.001), more subjective support (Beta = 0.124, *t* = 8.493, *p* < 0.001), and higher EPQ-P score (Beta = 0.183, *t* = 16.891, *p* < 0.001) were associated with higher QoL (Table 4).

**Table 4:** Multivariate analysis for variables associated with EQ-VAS.

	**Unstandardized coefficients**		**Standardized coefficients**		
**Variables**	***B***	**SE**	**Beta**	***t***	**Sig**.
Being homeless	−4.149	0.29	−0.156	−14.29	<0.001
Sex (Female)	−5.559	0.286	−0.2	−19.406	<0.001
Age	0.265	0.013	0.219	20.112	<0.001
**SSRS**				
Objective support	0.816	0.044	0.283	18.419	<0.001
Subjective support	0.261	0.05	0.077	5.171	<0.001
Social support	0.708	0.083	0.124	8.493	<0.001
**Eysenck Personality Questionnaire**				
Psychoticism	0.254	0.015	0.183	16.891	<0.001
Extroversion	−0.058	0.015	−0.047	−3.941	<0.001
Neuroticism	−0.135	0.013	−0.119	−10.195	<0.001
Lie	−0.239	0.017	−0.156	−13.756	<0.001

## Discussion

Our study systematically compared the sociodemographic characteristics, social support, and QoL between homeless and non-homeless patients with schizophrenia. To the best of our knowledge, this is the first study of its kind in China. The results showed that homeless patients were less likely to obtain social support. Although no difference in the EQ-5D health index was observed between the two groups, homeless patients experienced more general anxiety, depression, and physical pain as evaluated by the EQ-VAS.

The results indicated considerable sociodemographic differences between homeless and non-homeless patients. Most patients included were of Han ethnicity, accounting for 89–95.2% of the participants. The proportion of ethnic minorities among homeless patients was higher than that among non-homeless. This could be because, by the end of 2016, 99.5% of the local residents were of Han ethnicity, while 0.5% were ethnic minorities. Furthermore, the homeless patients were more likely to live primarily outside the city (87%), which is different to the findings on homeless patients with schizophrenia in other countries (23). This result may be related to the fact that Xiangtan is a major agricultural city, and a large section of the population lives in the countryside. And we recorded their primary hometown in the Hukou system, which were different from the methods in studies from other countries.

Our study found that most patients included were not married, with an unmarried rate of over 56.6%. The homeless patients had a higher rate of being divorced or widowed (13.7 vs. 17.7%). Regardless of cultural norms and socio-economic contexts, marriage can provide important benefits to persons with schizophrenia through stronger social networks and support and a better QoL (24). However, cross-cultural comparative research has documented lower rates of marriage and higher rates of separation and divorce among persons with schizophrenia when compared to the general population (25). First, patients with schizophrenia encounter barriers to forming matrimonial alliances because the most opportune time for courtship and marriage often corresponds with an early, insidious onset of the illness (26). Second, among those who were married, the poorer prognosis of the illness and lower socio-economic status were shown to be predictors of divorce and separation (27). In this study, although only a small proportion of homeless patients had stable jobs, the previous employment rate of homeless patients (72.6%) was much higher than that of the non-homeless (52.4%). The lack of social acceptance of patients with schizophrenia could be one of the main obstacles to marriage or employment.

The average age of homeless patients was higher compared with previously reported literatures on homeless people. This may due to the earlier onset of schizophrenia and a relatively lower education level in our sample (5, 8, 28). Patients with schizophrenia may be supported by their relatives during the early stages of the disease, but as the condition worsens, their families experience heavy economic burden, slowly eroding treatment confidence, and leading to abandonment (29). The results also showed that almost half of the patients were smokers (47.3–49.7%) (30), which was markedly higher than the general population (20%) (31), but lower than in the report by Torchalla et al. (80.8%) (32).

The homeless patients were less likely to receive social support. There is also a need to provide supported accommodation for people whose social skills are limited by severe psychiatric and intellectual disabilities, such as through required skill training and psychosocial and vocational training (23, 33). The Chinese government has tried to provide more social security for those with mental illness by building a prevention and treatment network, releasing favorable health care and medical insurance policies, providing financial aids for their guardians, establishing their rights and obligations, and granting tax reductions for enterprises hiring them. The Mental Health Law of the People's Republic of China (34) was issued on May 1st, 2013, which provides measures for the administration of relief for the homeless and beggars living in the city, along with various local supporting laws and regulations to ensure their implementation.

The QoL of patients with schizophrenia is affected by many factors, such as housing, healthcare, basic human rights, vocational skills training, and other domains (33, 35). Housing is crucial for improving one's QoL (35). Our study found that in patients with schizophrenia, the QoL of non-homeless patients may be better than that of homeless patients. An important difference is relatively stable housing. Our study also found that the QoL of patients with schizophrenia was generally low; the highest EQ-5D health index was 0.848, and the median was 0.768. They commonly felt worried, sad, or unhappy (50.7%), and experienced pain or discomfort (54.8%). Fond et al. (36) also reported that more than half of the patients (51.5%) reported moderate to extreme physical pain, while only 2.7% were administered analgesic drugs; physical pain is highly frequent and undertreated in homeless participants with mental illness. Attention should also be paid to ex-homeless women; a recent study reported that ex-homeless women with schizophrenia were more exposed to verbal, physical, and sexual violence, which may strongly affect their mental and physical health as well as QoL and increase their suicide risk (37). Our results also indicated that homeless patients with schizophrenia tended to have more abnormal levels of psychoticism and neuroticism than non-homeless patients, which indicate higher levels of antisocial behavior and emotional distress, respectively. However, they had lower scores in the dimensions of extroversion and dissimulation. Lower scores on the Lie scale indicate reduced concern with socially desirable behavior. These results may indicate that the homeless are more poorly adjusted psychologically than the non-homeless. There is existing literature that has found that high neuroticism and psychoticism and low extroversion are associated with poorer life outcomes (38–40).

There are several limitations to this study. First, we examined a sample of inpatients, rather than a general sample of outpatients or the homeless. Second, hospital costs and length of stay were not included in the study. Previous studies demonstrated that homeless patients were hospitalized longer and had excess costs (41, 42). Third, some important factors were not considered, such as material dependence, intellectual disability, and other mental health problems. Material dependence is an important factor among homeless individuals with mental illness as reported by developed countries (43, 44). Finally, the population was predominantly from the city of Xiangtan in the Hunan Province and the results may not be generalized to other populations.

Homeless patients with schizophrenia were less likely to have social support, and there is a need for more supported accommodation and skills training for patients limited by severe psychiatric diseases. The patients' QoL was relatively low; they experienced general anxiety, depression, and physical pain. This indicates that more attention must be given to the emotional state of patients while providing active assistance, and better medical treatment must be provided to reduce their physical pain appropriately, and to improve their QoL.

## Data availability statement

The raw data supporting the conclusions of this article will be made available by the authors, without undue reservation.

## Ethics statement

The studies involving human participants were reviewed and approved by the Ethic Committees of the Fifth People's Hospital of Xiangtan. Written informed consent to participate in this study was provided by the participants' legal guardian/next of kin.

## Author contributions

JindC and JH: conceptualization, methodology, and supervision. JH: formal analysis and writing the original draft. JinlC: data collection. ZT and YS: data analysis. HS and SL: help in data collection and validation.

## Funding

The study was supported by Bao'an Traditional Chinese Medicine Development Foundation of Shenzhen city, Guangdong Province (No. 2020KJCX-KTYJ-139) and Sanming Project of Medicine in Shenzhen (No. SZZYSM202106009).

## Conflict of interest

The authors declare that the research was conducted in the absence of any commercial or financial relationships that could be construed as a potential conflict of interest.

## Publisher's note

All claims expressed in this article are solely those of the authors and do not necessarily represent those of their affiliated organizations, or those of the publisher, the editors and the reviewers. Any product that may be evaluated in this article, or claim that may be made by its manufacturer, is not guaranteed or endorsed by the publisher.

## Footnotes

^1^Available from: https://www.hudexchange.info/resource/4847/hearth-defining-chronically-homeless-final-rule/
